# Mapping the Progression of Atrial Fibrillation Substrate: Insights from Left Atrial Wall Thickness and High-Density Voltage Mapping

**DOI:** 10.3390/jcm15176608

**Published:** 2026-08-27

**Authors:** Etel Silva, Manuel González-Armayones, Iván Lobo-Torres, Rafael Fernández-Rivero, Saman Golmaryami, Ana María Solano-Mulero, Alejandro Pérez-Martín, Andrés García-Gámez, Juan Carlos García-Benítez, Lucas Cano-Calabria, Juan Fernández-Armenta

**Affiliations:** 1 Cardiology Department, Institute of Biomedical Research and Innovation of Cádiz (INiBICA), University Hospital Puerta del Mar, 11009 Cádiz, Spain; 2 Radiology Department, University Hospital Puerta del Mar, 11009 Cádiz, Spain

**Keywords:** atrial fibrillation, left atrial wall thickness, computed tomography, high-density voltage mapping, atrial remodeling, atrial cardiomyopathy

## Abstract

**Background/Objective:** Advances in cardiac computed tomography (CT) now allow high-resolution, three-dimensional reconstruction of the left atrium, enabling precise quantification of left atrial wall thickness (LAWT). However, the relationship between LAWT and electrophysiological properties, particularly as assessed by high-density bipolar voltage mapping, remains poorly defined. The objective was to evaluate left atrial structural remodeling in atrial fibrillation (AF) by integrating three-dimensional CT-derived LAWT maps with high-density bipolar voltage mapping, and to assess the relationship between structural remodeling and electrophysiological changes across AF phenotypes. **Methods:** In this single-center observational study, 60 patients (30 with paroxysmal AF and 30 with persistent AF) undergoing their first AF ablation were enrolled. Pre-procedural cardiac CT scans were analysed using a validated artificial intelligence (AI)-based method to generate three-dimensional LAWT maps. The left atrium was segmented into five regions (lateral, anterior, septal, posterior, and roof) excluding the pulmonary veins and left atrial appendage. High-density bipolar voltage maps were acquired using the CARTO system and a multipolar catheter. Global and regional LAWT and bipolar voltage values were compared between AF phenotypes. The association between global LAWT and bipolar voltage was assessed at the patient level using Pearson’s correlation coefficient, whereas regional comparisons and associations between LAWT and bipolar voltage were evaluated using linear mixed-effects models accounting for repeated measurements within patients. **Results:** Patients with persistent AF showed larger left atrial volume (119 ± 26 vs. 102 ± 31 mL; *p* = 0.024) and a trend toward greater CT-derived LAWT, with significant differences observed in selected atrial regions. Overall mean bipolar voltage and LAWT were 1.86 ± 0.59 mV and 1.45 ± 0.32 mm, respectively. No meaningful linear association was observed between these parameters (global Pearson r = −0.018), a finding that persisted across regions and AF phenotypes. No clinical factors were significantly associated with global LAWT, although heart failure and persistent AF showed a trend toward greater global wall thickness. **Conclusions:** Compared with paroxysmal AF, persistent AF was associated with a tendency toward increased left atrial wall thickness, consistent with more advanced structural remodeling. The lack of a meaningful correlation between wall thickness and bipolar voltage suggests that LAWT provides complementary structural information beyond electroanatomical mapping.

## 1. Introduction

Atrial fibrillation (AF) is the most common sustained arrhythmia, associated with increased risk of stroke, heart failure, and mortality. [1] Contemporary concepts recognize AF as both a consequence and a driver of atrial cardiomyopathy, a multifactorial disease process characterized by structural, electrical, and functional remodeling of the atrial myocardium. These alterations arise from the combined effects of aging, cardiovascular risk factors, and comorbidities, while AF itself further promotes progressive remodeling, establishing a bidirectional, self-perpetuating relationship between the arrhythmia and its underlying substrate [2,3]. Structural remodeling of the left atrium (LA)—driven by mechanisms including cardiomyocyte hypertrophy, extracellular matrix expansion, and fibrosis—may therefore contribute to AF progression and influence treatment response [4].

Left atrial wall thickness (LAWT), measurable with high-resolution cardiac computed tomography (CT), has emerged as a potential surrogate marker of atrial remodeling [5,6]. While low-voltage areas (LVAs) identified by electroanatomical mapping are associated with ablation outcomes, the relationship between LAWT and bipolar voltage remains incompletely understood, with inconsistent findings across studies [7,8,9].

This study aims to characterize atrial substrate differences between paroxysmal and persistent AF phenotypes and to investigate the association between CT-derived LAWT and high-density bipolar voltage mapping across predefined atrial regions.

## 2. Methods

### 2.1. Patient Population

This observational, descriptive study included patients over 18 years with a history of paroxysmal or persistent atrial fibrillation undergoing their first AF ablation at Hospital Puerta del Mar. All patients had symptomatic AF with an indication for ablation in accordance with current European Society of Cardiology (ESC) guidelines. Persistent AF was defined as any episode lasting continuously for more than 7 days.

The study was approved by the local ethics committee and conducted in accordance with the Declaration of Helsinki. Written informed consent was obtained from all participants.

### 2.2. Image Processing/Wall Thickness Analysis

Cardiac CT scans were acquired within 48 h before the ablation procedure. LAWT was computed with ADAS3D (Galgo Medical, v2.14.0-beta.5, 7 May 2025). LA wall contours—excluding the LA appendage and the mitral valve—were automatically delineated by an artificial-intelligence algorithm, yielding a 3D LAWT map for each patient. As part of the semi-automated workflow, minor manual refinement of the epicardial border was performed in virtually all patients to correct small segmentation inaccuracies before generation of the final three-dimensional LAWT map. Pulmonary veins were manually cropped when required, and the quality of the automated wall tracking was reviewed blinded to outcomes before analysis, following the general approach described previously for AI-based LAWT mapping [10].

The resulting 3D LAWT models were imported into the electroanatomical navigation system (CARTO3, Biosense Webster) and co-registered with the high-density bipolar voltage maps to ensure both thickness and voltage were represented in the same coordinate space. After the procedure, the integrated LAWT–electroanatomical mapping (EAM) dataset was exported for offline analyses. Consistent with prior protocols in our cohort, pulmonary veins and the left atrial appendage were excluded from quantitative analyses.

**Figure 1:**
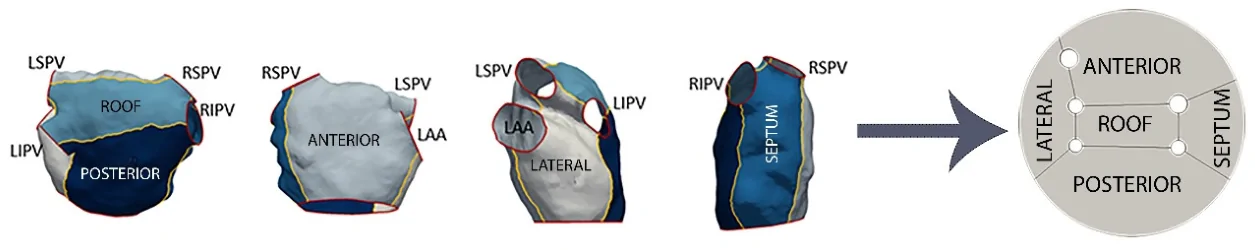
Standardized planar representation of the left atrium showing the five predefined anatomical regions used for analysis: lateral, anterior, septal, posterior, and roof. Regional left atrial wall thickness and co-registered bipolar voltage values were computed within each segment for subsequent analysis.

For regional analyses, the left atrium was partitioned using the Fast Quasi-Conformal Regional Flattening approach of Núñez-García et al., [11] generating a standardized planar representation from which we defined five anatomical regions: lateral, anterior, septal, posterior and roof (Figure 1). Regional LAWT and co-registered bipolar voltage were then computed over these segments for subsequent statistical analysis.

### 2.3. Electrophysiology Study

All ablation procedures were performed under uninterrupted oral anticoagulation. Procedures were conducted under general anesthesia or deep sedation, depending on the patient’s clinical characteristics and institutional protocol. Vascular access was obtained via one or two right femoral venous punctures, performed under ultrasound guidance. Transseptal puncture was guided by either transesophageal or intracardiac echocardiography. High-density bipolar voltage mapping was performed before ablation during sinus rhythm using CARTO3 and a multipolar catheter (PentaRay^®^, Biosense Webster, Diamond Bar, CA, USA). Patients presenting in atrial fibrillation underwent electrical cardioversion before voltage mapping. In patients undergoing general anesthesia, mechanical ventilation was performed using a high-frequency, low-tidal-volume protocol to minimize respiratory motion, whereas respiratory gating was used in patients undergoing conscious sedation. The bandpass filter was set to 16–500 Hz and bipolar electrograms (EGMs) were displayed at sweep speeds of 100–200 mm/s and 0.1 mm/mV. Adequate endocardial contact was confirmed by stable bipolar electrograms, proximity to the geometry surface and the Tissue Proximity Indicator (TPI). A fill threshold of 5 mm was required to obtain enough density.

### 2.4. Statistical Analysis

A total of 60 patients were included, comprising 30 patients with paroxysmal AF and 30 with persistent AF. Categorical variables are presented as counts and percentages. Continuous variables are presented as mean ± standard deviation or median [interquartile range], as appropriate. Baseline characteristics were compared between AF phenotypes using Student’s *t*-test or the Wilcoxon rank-sum test for continuous variables, and the chi-square test or Fisher’s exact test for categorical variables, as appropriate.

CT-derived left atrial wall-thickness measurements were spatially matched to high-density electroanatomical mapping points. Point-level data were used to describe the overall distributions of wall thickness and bipolar voltage and to calculate descriptive point-level correlations in the overall cohort and after stratification by AF phenotype. However, individual mapping points were not treated as statistically independent observations in inferential analyses. For global analyses, point-level measurements were aggregated into one mean bipolar voltage and one mean wall-thickness value per patient. For regional analyses, one mean bipolar voltage and one mean wall-thickness value were calculated for each patient and predefined anatomical region.

The global association between wall thickness and bipolar voltage was evaluated at the patient level using Pearson’s correlation coefficient and univariable linear regression, with mean bipolar voltage as the dependent variable and mean wall thickness as the explanatory variable. Global differences between paroxysmal and persistent AF were assessed using unadjusted patient-level linear regression models with AF phenotype as the explanatory variable. Results are reported as estimated mean differences with 95% confidence intervals (CIs).

Regional differences in bipolar voltage and wall thickness were evaluated using linear mixed-effects models with anatomical region as a fixed effect and patient as a random intercept. Nested models with and without the regional fixed effect were compared using likelihood-ratio tests. Models used for fixed-effect comparisons were fitted by maximum likelihood. When the overall regional effect was significant, pairwise comparisons between anatomical regions were obtained from estimated marginal means and adjusted using Tukey’s method.

Differences between paroxysmal and persistent AF across anatomical regions were evaluated using mixed-effects models including AF phenotype, anatomical region, and their interaction as fixed effects, with patient as a random intercept. The interaction term was used to determine whether between-phenotype differences varied across anatomical regions. Comparisons between AF phenotypes within each region were derived from estimated marginal means and adjusted for multiple comparisons using the Holm method.

The regional association between wall thickness and bipolar voltage was evaluated using a mixed-effects model with regional mean bipolar voltage as the dependent variable, regional mean wall thickness and anatomical region as fixed effects, and patient as a random intercept. A wall thickness-by-region interaction was additionally tested to assess whether this association differed across anatomical regions.

The global association between left atrial volume and mean CT-derived wall thickness was evaluated at the patient level using univariable linear regression. The regional association between left atrial volume and wall thickness was evaluated using a mixed-effects model based on patient-region averages. Left atrial volume, anatomical region, and their interaction were included as fixed effects, with patient as a random intercept. The interaction term was used to assess whether the association between left atrial volume and wall thickness differed across anatomical regions. Global and region-specific estimates were expressed per 10 mL increase in left atrial volume. Region-specific slopes were derived from the interaction model and adjusted for multiple comparisons using the Holm method.

Point-based low-voltage burden was calculated for each patient as the percentage of valid mapping points with bipolar voltage < 0.5 mV. This exploratory patient-level measure did not represent geometrically quantified low-voltage surface area. Low-voltage burden was summarized as median [interquartile range (IQR)] and compared between AF phenotypes using the Wilcoxon rank-sum test.

Differences in global wall thickness according to 12-month AF recurrence were assessed using Welch’s two-sample *t*-test because of the unequal group sizes and without assuming equal variances. The Wilcoxon rank-sum test was performed as a sensitivity analysis.

Exploratory patient-level univariable linear regression analyses were performed to investigate clinical factors potentially associated with global CT-derived wall thickness. Evaluated variables included AF phenotype, age, sex, body mass index, hypertension, diabetes, dyslipidemia, obstructive sleep apnea, smoking status, heart failure, left ventricular ejection fraction, left atrial anteroposterior diameter, left atrial volume, and left atrial volume index. Continuous variables were retained as continuous measures and rescaled to clinically interpretable increments. Left atrial volume index was calculated as CT-derived left atrial volume divided by body surface area. Given the limited cohort size and the absence of statistically significant univariable associations, no multivariable model was constructed.

Analyses were performed using complete available cases without imputation of missing data. All tests were two-sided, and a *p* value < 0.05 was considered statistically significant. Statistical analyses were performed using R version 4.5.2, including the lme4, lmerTest, and emmeans packages.

## 3. Results

### 3.1. General Characteristics

A total of 60 patients undergoing first-time atrial fibrillation ablation were included, comprising 30 patients with paroxysmal AF and 30 with persistent AF (Table 1). The mean age of the overall cohort was 58 ± 11 years, and 44 patients (73.3%) were male. Hypertension was the most prevalent comorbidity, affecting 31 patients (51.7%), followed by dyslipidemia in 18 (30.0%) and diabetes mellitus in 10 (16.7%). The mean body mass index was 30 ± 5 kg/m^2^. All electroanatomical maps were acquired in sinus rhythm; nine patients who presented in atrial fibrillation underwent electrical cardioversion before mapping.

Compared with patients with paroxysmal AF, those with persistent AF had a lower left ventricular ejection fraction (58 ± 11% vs. 65 ± 6%; *p* = 0.027), a higher body mass index (31 ± 5 kg/m^2^ vs. 28 ± 4 kg/m^2^; *p* = 0.038), a larger left atrial anteroposterior diameter (44 ± 6 vs. 38 ± 6 mm; *p* < 0.001), and a greater left atrial volume (119 ± 26 vs. 102 ± 31 mL; *p* = 0.024). No other baseline clinical characteristics differed significantly between AF phenotypes.

### 3.2. Atrial Wall Thickness and Bipolar Voltage: Global and Regional Analysis

After excluding points with bipolar voltage <0.01 or >8 mV to minimize potential artifacts, 26,305 spatially matched electroanatomical points were retained for descriptive analyses and for the calculation of patient-level global and regional averages. Of these, 11,624 corresponded to patients with paroxysmal AF and 14,681 to patients with persistent AF. Supplementary Table S1 presents the number of mapping points acquired per patient and across the predefined left atrial regions.

Individual mapping points were used to calculate patient-level global and regional averages and were not treated as independent observations in inferential analyses. The resulting analytical dataset comprised 60 patient-level global observations and 295 patient-region observations.

A descriptive point-level correlation analysis showed no meaningful linear relationship between bipolar voltage and wall thickness (Pearson’s r = −0.018). At the patient level, mean bipolar voltage was 1.86 ± 0.59 mV and mean CT-derived left atrial wall thickness was 1.45 ± 0.32 mm. Mean wall thickness was not significantly associated with mean bipolar voltage (β = 0.203 mV per 1 mm increase in wall thickness; 95% CI, −0.270 to 0.675; *p* = 0.395).

In the regional mixed-effects analysis, regional mean LAWT was not significantly associated with regional mean bipolar voltage after accounting for anatomical region and repeated measurements within patients (β = −0.120 mV/mm; *p* = 0.183). There was no evidence that this association differed by anatomical region (*p* for interaction = 0.388).

At the patient level, left atrial volume was not significantly associated with global mean CT-derived left atrial wall thickness (β = −0.012 mm per 10 mL increase in left atrial volume; 95% CI, −0.041 to 0.016; *p* = 0.388). However, this association differed significantly across anatomical regions (*p* for interaction = 0.0005). Greater left atrial volume was associated with increased lateral wall thickness, with an estimated increase of 0.056 mm per 10 mL increase in left atrial volume (95% CI, 0.017–0.095; Holm-adjusted *p* = 0.026), whereas no significant associations were observed in the anterior, septal, roof, or posterior walls.

Bipolar voltage differed significantly across anatomical regions in the mixed-effects model (global *p* < 0.001), ranging from 1.40 ± 0.53 mV in the septal wall to 2.11 ± 0.76 mV in the roof. Tukey-adjusted comparisons showed that septal voltage was significantly lower than in all other regions, while no significant differences were observed among the roof, posterior, and lateral walls. Wall thickness also varied significantly by region (global *p* < 0.001), with the lateral wall being the thickest (1.85 ± 0.53 mm) and significantly thicker than all other regions. The posterior wall showed the lowest mean thickness (1.41 ± 0.37 mm), although it did not differ significantly from the anterior, septal, or roof walls.

### 3.3. Atrial Wall Thickness and Bipolar Voltage According to AF Phenotype

Stratified analysis by AF phenotype demonstrated broadly similar regional distributions of bipolar voltage and wall thickness in both groups. Bipolar voltage differed significantly across anatomical regions in both paroxysmal and persistent AF (global *p* < 0.001 for both), with the septal wall showing the lowest mean values and the roof the highest. Wall thickness also differed significantly by region in both groups (global *p* < 0.001 for both), with the lateral wall being significantly thicker than all other regions.

Stratified point-level correlation analyses showed no meaningful linear relationship between bipolar voltage and wall thickness in either AF phenotype (Pearson’s r = −0.019 in paroxysmal AF and r = −0.001 in persistent AF).

As an exploratory patient-level measure of low-voltage substrate, point-based low-voltage burden was defined as the proportion of valid mapping points with bipolar voltage < 0.5 mV. The median burden in the overall cohort was 16.2% [IQR 10.7–23.8]. Patients with persistent AF showed a numerically higher burden than those with paroxysmal AF (16.9% [10.8–27.9] vs. 13.2% [10.8–19.2]), although the difference was not statistically significant (*p* = 0.186).

At the patient level, patients with persistent AF showed lower mean bipolar voltage than those with paroxysmal AF (1.73 ± 0.63 vs. 1.98 ± 0.51 mV; mean difference, −0.246 mV; 95% CI, −0.545 to 0.052; *p* = 0.103) and greater mean CT-derived left atrial wall thickness (1.51 ± 0.36 vs. 1.38 ± 0.27 mm; mean difference, 0.132 mm; 95% CI, −0.033 to 0.297; *p* = 0.115), although neither global difference reached statistical significance (Table 2).

**Figure 2:**
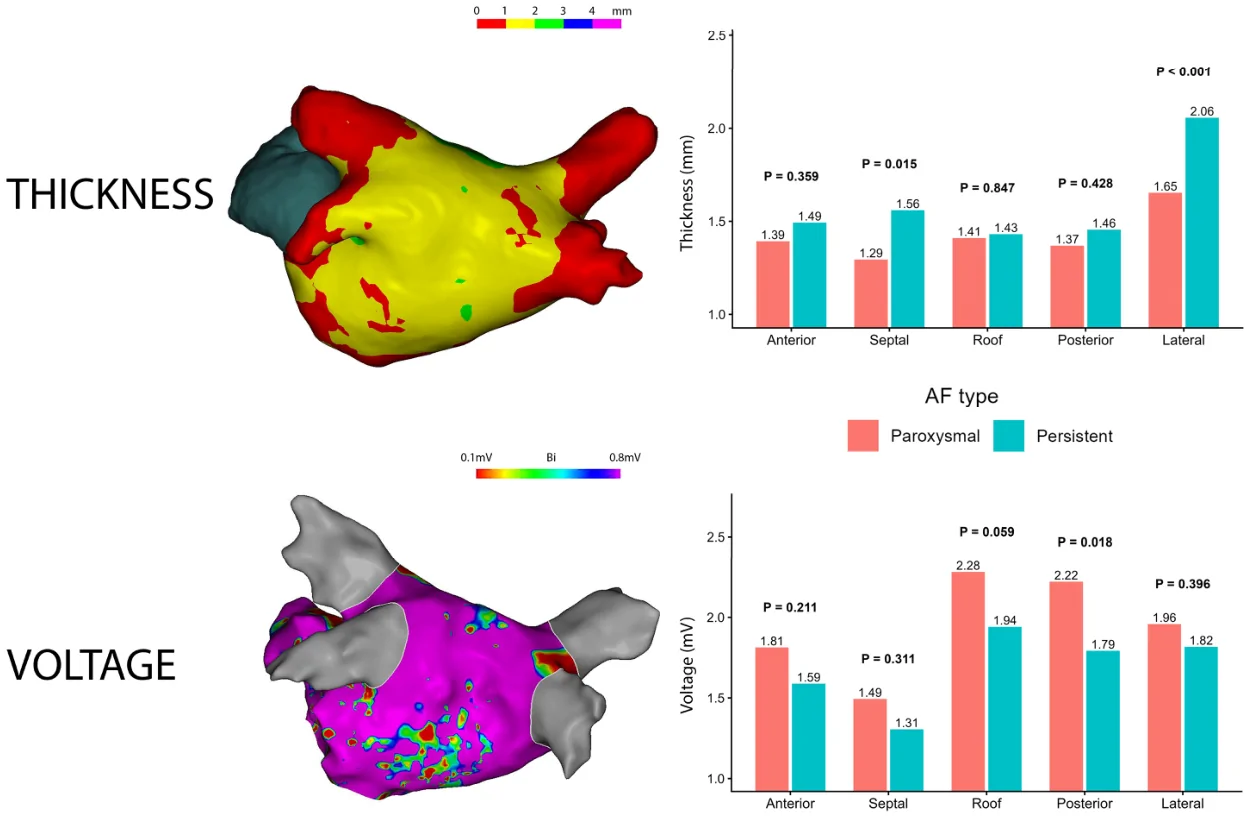
Regional distribution of left atrial wall thickness (superior panel) and bipolar voltage (inferior panel) across the predefined left atrial anatomical segments.

**Figure 3:**
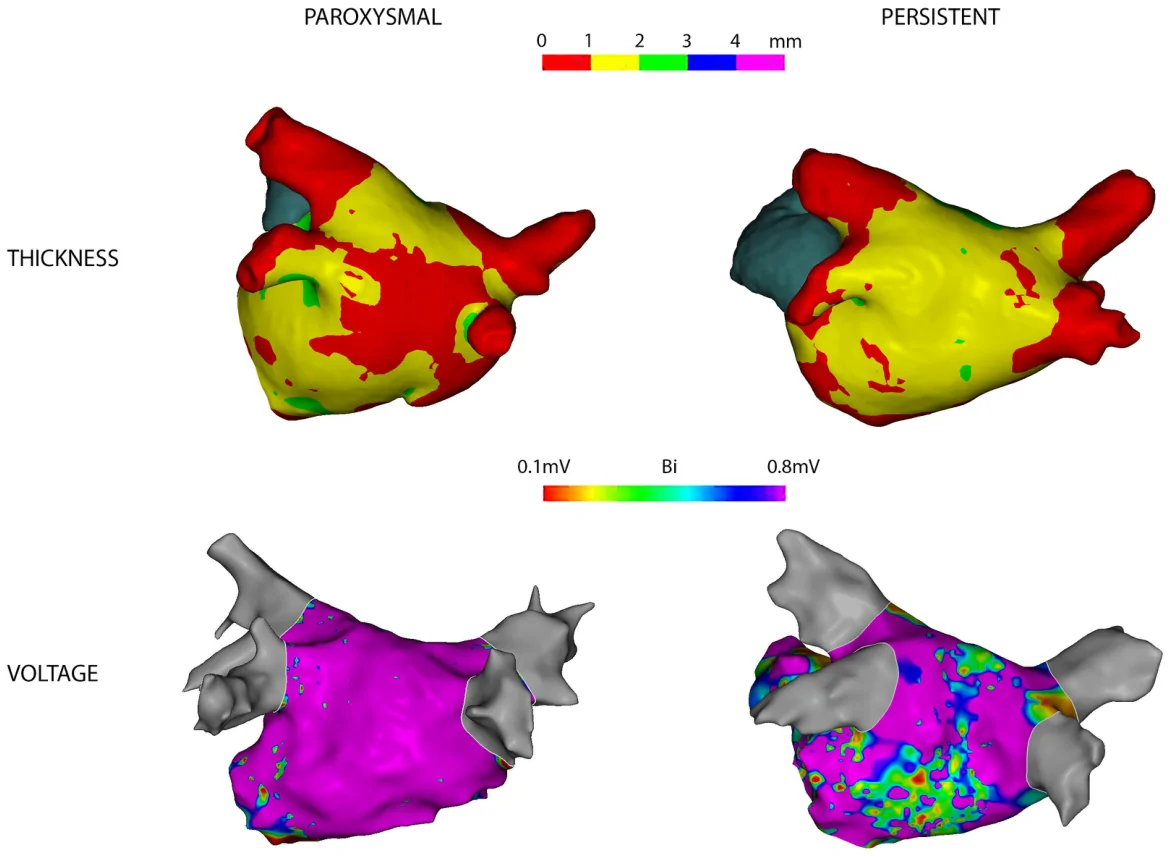
Representative examples of paroxysmal and persistent atrial fibrillation showing co-registered left atrial wall thickness and bipolar voltage maps.

In the regional mixed-effects analysis, bipolar voltage was lower in persistent AF across all anatomical regions, reaching statistical significance only in the posterior wall after Holm correction. Wall thickness was greater in persistent AF across all regions, with significant differences in the septal and lateral walls (Figure 2). The reduction in bipolar voltage observed in persistent AF was relatively uniform across anatomical regions, whereas differences in wall thickness varied significantly by region. Figure 3 provides representative examples from patients with paroxysmal and persistent AF, displaying the corresponding left atrial wall thickness and bipolar voltage maps.

FU: During follow-up, 12 patients (20%) experienced AF recurrence. Mean left atrial wall thickness did not differ between patients with and without recurrence (1.43 ± 0.21 mm vs. 1.45 ± 0.35 mm, *p* = 0.807).

### 3.4. Univariable Analysis of Factors Associated with Global Wall Thickness

Patient-level univariable linear regression analyses were performed to evaluate the association between global CT-derived LAWT, as specified in the Methods. No clinical factors were significantly associated with global LAWT. Heart failure showed the strongest association with greater wall thickness (β = 0.323 mm, 95% CI −0.014 to 0.660; *p* = 0.06), followed by persistent AF compared with paroxysmal AF (β, 0.132 mm; 95% CI, −0.033 to 0.297; *p* = 0.115). Consequently, no multivariable model was constructed.

## 4. Discussion

In this study, we provide a detailed characterization of atrial structural remodeling using CT-derived LAWT integrated with high-density bipolar voltage mapping. The main findings are as follows: First, patients with persistent AF showed numerically greater global LAWT and lower global bipolar voltage than those with paroxysmal AF, although neither global difference reached statistical significance. Regional analyses demonstrated greater LAWT in the septal and lateral walls and lower bipolar voltage in the posterior wall in persistent AF. Second, no meaningful correlation was observed between regional LAWT and bipolar voltage, indicating that tissue thickening and electrical attenuation represent distinct and only partially overlapping aspects of atrial remodeling. Third, LA volume was not associated with global LAWT, although a region-specific positive association was observed in the lateral wall. Taken together, these findings suggest that AF progression is accompanied by parallel but dissociated structural and electrophysiological changes—namely, wall thickening and voltage reduction—that evolve independently and cannot be assessed by high-density voltage mapping alone. This dissociation between structural hypertrophy and electrical remodeling aligns with the current concept of atrial cardiomyopathy, which recognizes atrial disease as a multidimensional process involving structural, electrical, and functional abnormalities that do not necessarily evolve in parallel [2].

LAWT is becoming increasingly accessible through novel CT image processing and machine learning technologies, evolving from focal measurements at predefined sites to semi-automated three-dimensional reconstructions of the entire left atrial wall [12,13]. LA hypertrophy is emerging as a novel marker of structural remodeling along the AF continuum [14].

Previous pathological and imaging studies have yielded inconsistent findings regarding the relationship between LAWT and AF progression. It has often been suggested that atrial remodeling ultimately leads to wall thinning. In support of this hypothesis, Platonov et al. examined posterior left atrial specimens obtained from routine autopsies and reported that patients with a history of AF had significantly thinner posterior atrial walls compared with those without AF [15]. Some studies, such as Takahashi et al., have reported significantly thicker LA walls in patients with AF compared with healthy controls, with a stepwise increase from paroxysmal to persistent AF; in contrast, other studies have suggested the opposite pattern, with wall thinning in more advanced stages of AF [6,16,17]. More recent imaging studies based on three-dimensional reconstructions of the LA wall, such as that of Taormina et al., are consistent with our findings, reporting greater LAWT in persistent compared with paroxysmal AF [18].

Recent studies have identified LAWT as an independent predictor of long-term AF recurrence following pulmonary vein isolation [12,19]. Notably, previous studies have reported no significant association between global LAWT and left atrial volume index [12]. Similarly, left atrial volume was not associated with global mean LAWT in our patient-level analysis. However, the relationship was region-dependent, with a significant positive association observed only in the lateral wall. These findings suggest that LAWT reflects a regionally heterogeneous manifestation of structural remodeling and may provide information beyond traditional global markers such as chamber volume.

The presence of low-voltage areas (LVAs) is a key feature of AF progression and an established predictor of AF ablation outcomes. However, as shown in the ERASE trial, LVAs were identified in only approximately 35% of patients undergoing ablation for persistent AF, suggesting that voltage mapping alone may underestimate the true extent of the structural substrate [20]. Efforts to spatially correlate LAWT with local tissue voltage have yielded conflicting findings. Nakatani et al. reported atrial thinning as an independent predictor of LVAs, with wall thickness < 1.9 mm associated with more extensive low-voltage regions, likely reflecting myocyte loss and replacement fibrosis [8]. In contrast, Takahashi et al. found that increased thickness at the pulmonary vein-LA junction was associated with higher bipolar voltage, suggesting that hypertrophied tissue in this region increases local electrical activity and facilitates AF maintenance. More recently, Li et al. reported a positive correlation between posterior wall thickness and the extent of LVAs, challenging a simple “thinner-equals-more-fibrotic” paradigm and highlighting the complexity of the structure–voltage relationship [21]. In line with this, Gomes et al. demonstrated a moderate correlation between mean LAWT and the fibrosis burden quantified by LGE-CMR, supporting LAWT as a macroscopic marker of structural remodeling rather than a direct surrogate of local voltage abnormalities [12]. In contrast, both Taormina et al. and the present study failed to identify a robust segment-by-segment correlation between regional LAWT and bipolar voltage, despite observing clear differences in LAWT between AF phenotypes [18]. Taken together, these observations suggest that LVAs, increased LAWT and LGE-CMR-defined fibrosis represent related but only partially overlapping dimensions of atrial remodeling, each providing complementary rather than redundant information.

In the healthy twin cohort reported by Whitaker et al., hypertension was associated with an increase in LA volume but not in LAWT, and other AF-predisposing conditions did not modify LAWT in subjects without structural heart disease [22]. Consistent with these observations, no clinical factor was significantly associated with global LAWT in our cohort. Heart failure and persistent AF showed the largest effect estimates for greater global wall thickness, although neither association reached statistical significance. Taken together with the region-specific association between left atrial volume and lateral wall thickness, these findings suggest that the determinants of LAWT may vary across anatomical regions and may not be adequately captured by global structural markers.

## 5. Limitations

Several limitations should be acknowledged. First, this single-centre observational study included a relatively small number of patients. Although mixed-effects models were used to account for repeated regional measurements within individuals, the limited patient-level sample size reduced the precision of effect estimates and constrained interaction, subgroup, and multivariable analyses, potentially limiting the generalisability of our findings. Second, the cross-sectional design, with CT imaging acquired at a single pre-procedural time point, precludes any causal inferences regarding the temporal relationship between LAWT, LVAs and AF progression. Third, LAWT was derived from a specific semi-automated software platform (ADAS3D); although representative of current clinical practice, measurement variability and potential inaccuracies in CT-EAM co-registration cannot be entirely excluded. Fourth, the LA was segmented into five relatively broad anatomical regions; while this facilitates inter-patient comparisons, it may be too coarse to detect focal associations between LAWT and voltage at specific sites such as the pulmonary vein–LA junction or the left atrial ridge. Fifth, complementary markers of structural remodeling such as late gadolinium enhancement cardiac magnetic resonance (LGE-CMR) fibrosis or histopathological data were not incorporated, limiting the ability to further characterize the relationship between LAWT, voltage, and underlying tissue composition. Finally, analyses of clinical factors associated with global LAWT were exploratory and limited to univariable models; therefore, residual confounding cannot be excluded, and the absence of statistically significant associations should not be interpreted as evidence of no effect. Larger multicenter studies with comprehensive multivariable modeling will be required to identify the clinical determinants of increased LAWT.

## 6. Conclusions

In this study, we integrated CT-derived left atrial wall thickness maps with high-density bipolar voltage mapping to characterize atrial remodeling across the AF spectrum. Persistent AF was associated with numerically greater global LAWT and significantly greater LAWT in selected atrial regions compared with paroxysmal AF, a pattern consistent with more advanced structural remodeling. In contrast, bipolar voltage showed no meaningful association with wall thickness at either the global or regional level, indicating that voltage mapping alone does not reliably reflect underlying LAWT.

These findings suggest that three-dimensional CT-based LAWT reconstructions provide structural information that is complementary to electroanatomical voltage mapping. LAWT assessment may therefore offer incremental value for risk stratification and procedural planning beyond traditional markers such as chamber size and low-voltage areas. Larger multicenter studies with longitudinal follow-up are needed to determine how best to incorporate LAWT into personalized strategies for AF ablation and long-term management.

## Figures and Tables

**Table 1:** Baseline clinical and imaging characteristics of the study population.

	All Patients (n = 60)	Paroxysmal AF (30)	Persistent AF (30)	*p*
Male sex, n (%)	44 (73.3%)	22 (73.3%)	22 (73.3%)	>0.9
Age	58 ± 11	56 ± 13	60 ± 8	0.59
LVEF	61 ± 10%	65 ± 6	58 ±11	0.027
Hypertension	31 (52%)	15 (50%)	16 (53%)	>0.9
Diabetes	10 (17%)	2 (6.7%)	8 (27%)	0.081
Dyslipidemia	18 (31%)	9 (30%)	9 (30%)	>0.9
BMI (kg/m^2^)	30 ± 5	28 ± 4	31 ± 5	0.038
OSAS	5 (8.3%)	2 (6.7%)	3 (10%)	>0.9
Smoking	6 (10%)	3(10%)	3 (10%)	>0.9
Heart failure	6 (10%)	2 (6.7%)	4 (17.4%)	0.11
LA Diameter (AP)	41 ± 7	38 ± 6	44 ± 6	<0.001
LA volume (mL)	110 ± 30	102 ± 31	119 ± 26	0.024

BMI, body mass index; LA, left atrium; LVEF, left ventricular ejection fraction; OSAS, obstructive sleep apnea syndrome; AP, anteroposterior; AF, atrial fibrillation.

**Table 2:** Global electroanatomical characteristics according to AF phenotype.

Variable	Overall	Paroxysmal AF	Persistent AF	Mean Difference (Persistent–Paroxysmal)	95% CI	*p*
Mean bipolar voltage (mV)	1.86 ± 0.59	1.98 ± 0.51	1.73 ± 0.63	−0.246	−0.545 to +0.052	0.103
Mean CT-derived LAWT (mm)	1.45 ± 0.32	1.38 ± 0.27	1.51 ± 0.36	+0.132	−0.033 to +0.297	0.115

Values are presented as patient-level mean ± SD. Mean differences and 95% confidence intervals were estimated using unadjusted linear models, with paroxysmal AF as the reference group. CI, confidence interval; CT, computed tomography; AF, atrial fibrillation.

## Data Availability

The data supporting the findings of this study are available from the corresponding author upon reasonable request, subject to applicable ethical and institutional restrictions.

## Supplementary Materials

The following supporting information can be downloaded at https://www.mdpi.com/article/10.3390/jcm15176608/s1, Table S1. Overall and regional distribution of electroanatomical mapping points.
