# Supplementary material for: Mapping the Progression of Atrial Fibrillation Substrate: Insights from Left Atrial Wall Thickness and High-Density Voltage Mapping
Source: J Clin Med. 2026 Aug 27;15(17):6608. doi: 10.3390/jcm15176608 (PMC13566029; doi:10.3390/jcm15176608)
Supplement: Supplementary file 1 [file jcm-15-06608-s001.zip › jcm-4386015-supplementary.pdf]

Supplementary Table S1. Overall and regional distribution of electroanatomical mapping points

Region	Patients, n	Mapping points, n	Mean ± SD	Median [IQR]
All regions	60	26,305	438.4 ± 225.1	361 [296–572]
**Distribution by anatomical region**				
Anterior	60	7,774	129.6 ± 95.5	98 [60–185]
Septal	59	3,569	60.5 ± 42.2	52 [31–80]
Roof	60	6,016	100.3 ± 61.0	84 [53–130]
Posterior	60	6,812	113.5 ± 77.7	96 [58–136]
Lateral	56	2,134	38.1 ± 31.9	34 [16–50]

Values represent the number of valid mapping points acquired per patient, overall and within each predefined anatomical region. IQR indicates interquartile range; SD, standard deviation.
